# Linking Pluripotency Reprogramming and Cancer

**DOI:** 10.5966/sctm.2015-0225

**Published:** 2016-09-20

**Authors:** Juan Manuel Iglesias, Juan Gumuzio, Angel G. Martin

**Affiliations:** ^1^ Synpromics Ltd., Edinburgh, United Kingdom; ^2^ StemTek Therapeutics, Bilbao, Spain

## Abstract

Tumor development and the generation of induced pluripotent stem cells are highly comparable processes with striking similarities. Cellular plasticity is inherent to tumor evolution, rendering cells that acquire a stem cell‐like phenotype, for which Sox2 activation has proved instrumental for the plastic acquisition of stemness properties in tumor cells. Understanding the molecular mechanisms underlying both events might uncover novel approaches for the development of anticancer therapeutics and constitute model systems for understanding tumor generation and ensuring the biosafety of cell‐based therapies. Stem Cells Translational Medicine *2017;6:335–339*

**Significance Statement**

Defining the molecular drivers of cancer stem‐like cells will provide avenues for the rational design of cancer treatment regimens aimed at cancer stem cells, which combined with conventional treatments targeting the bulk tumor cells, will provide novel therapies for cancer patients. Screening methods based on pluripotency transcriptional reporters would help to identify new drugs targeting cancer stem cells and cellular plasticity.

## Cancer Stem Cells and Pluripotency Reprogramming

The idea of a tumor being composed of nearly homogenous limitless replicating cells has been replaced by a concept in which tumors are composed of extremely heterogeneous cell populations. Subpopulations of cancer cells with enhanced tumorigenicity and bearing stemness features (defined as expression of stem cell genes), named cancer stem cells (CSCs) or tumor‐initiating cells (TICs), have been isolated from almost any type of tumor. TICs or CSCs share with pluripotent and adult stem cells gene networks that are essential for self‐renewal and pluripotency 1, 2. The reactivation of signaling pathways and genes essential for embryonic development during tumor progression and the ability of tumor cells to differentiate into many unrelated tissues and cell types, when placed in the right environment, highlight the link between pluripotency and cancer 3, 4, 5.

The generation of induced pluripotent stem cells (iPSCs) through genetic reprogramming of differentiated cells has created a wealth of knowledge on the mechanisms that control stemness properties 6. The similarities found between the process of pluripotency reprogramming and tumorigenesis are striking. Both processes require the expression or activation of oncogenes, the inactivation of tumor suppressor genes such as *p53* or *Rb* 7, 8, 9, 10, overriding of senescence and apoptotic barriers, extensive epigenetic changes, and a metabolic switch toward a glycolytic metabolism 11, 12, 13. Historical similarities also exist between the method used to generate iPSCs and the in vitro oncogenic transformation protocols used to identify the first oncogenes: target cells are transduced with a cocktail of viruses expressing a variety of suspected oncogenes (OKSM for pluripotency—Oct4, Klf4, Sox2, and c‐Myc). After extensive morphological selection of the resulting cell clones (colonies), some will be separated as established transformed lines (capable of generating tumors in immunosuppressed mice). When fibroblasts of identical genetic background were reprogrammed to iPSCs or transformed, their transcriptome changes were found to overlap significantly 11, although differences specific to the pluripotent state were present. Some of these common genes are responsible for the programs that make iPSCs tumorigenic, including response to wounding, lysosomes, and inflammation. Furthermore, transient expression of OKSM factors in vivo was capable of inducing dedifferentiation and generating pluripotent cells in multiple tissues; however, teratomas 14 and multiple dysplastic lesions 15 were readily observed in a multitude of organs. If OKSM induction was sustained long enough (more than 7 days), the tumors became independent of OKSM expression. These results highlight the relationship between the acquisition of pluripotency and tumor generation.

Pluripotency reprogramming has been finely mapped and occurs in two transcriptional waves. The initial wave occurs even in cells refractive to full reprogramming, with the second wave preceding the changes in DNA methylation necessary for stable pluripotency to be achieved 16. Transformation imposes activation of certain roadblocks that prevent the cell from fully reprograming into the pluripotent state. For example, applying the typical four‐factor reprogramming protocol to the breast carcinoma MCF7 cell line rendered clones that were only partially reprogrammed, with activation of just one of the endogenous pluripotency genes: *Sox2* 17. These clones were enhanced in cancer stem cell‐related features, but none were fully reprogrammed iPSCs. It is possible that this is why natural tumor formation has been found, but not in vivo natural reprogramming.

## Cellular Plasticity, Pluripotency, and Metastatic Invasion

The differentiated state tends to be a stable condition; however, cellular plasticity manifests under stress circumstances, such as tissue damage or infection. In such circumstances, short‐lived progenitors can dedifferentiate and acquire the ability to regenerate cells from other lineages, such as an entire mammary gland tree or intestinal crypts 18, 19, demonstrating that potency and plasticity are inherent cellular features.

Epithelial to mesenchymal transition (EMT) is a genetic program active during embryonic development and responsible for tissue remodeling and cellular motility. It is a well‐known process associated with fate determination in cells of epithelial lineage and therefore a candidate for playing a role during tumor progression. This process is activated during tumor invasion and metastasis as cells detach from the primary tumor mass, acquire mesenchymal motility properties, and migrate to distal locations. EMT is driven by transcription factors such as SNAI1/2, ZEB1/2, or TWIST1/2, resulting in enhanced migration and invasive potential of epithelial cells and is critical for the metastatic spread of epithelial tumors (a review is provided in 20). This process was demonstrated to potentiate the stem cell features of cancer cells 21, ensuring the colonization of distal sites and the generation of new tumors. Several mechanisms have been proposed to explain the EMT‐induced stemness features: the EMT inducer Zeb1 inhibits the expression of the miRNA200 family, upregulating the polycomb protein Bmi1 and inducing stemness 22.

In contrast, the reverse process of EMT, mesenchymal to epithelial transition (MET), is required to fully reprogram fibroblasts to iPSCs 23, apparently contradicting the role of EMT in generating stem cell properties. Moreover, the four canonical Yamanaka pluripotency factors transcriptionally block the EMT process: Sox2/Oct4 suppresses the EMT mediator SNAIL; c‐Myc downregulates transforming growth factor (TGF)‐β1 and TGF‐β receptor 2; and Klf4 induces epithelial genes, including E‐cadherin 23, 24, 25. In order to reconcile these observations, a sequential model is necessary. EMT is required first for tumor cell motility and invasion, and then MET takes over to regain self‐renewal and therefore the ability to colonize new niches. This possibility was demonstrated in breast cancer stem cells 26 and through the isolation of cell clones enriched in TICs showing either one of these programs and their modulation through expression of mesenchymal or epithelial promoting genes (SNAIL or E‐cadherin) 27. Cells expressing the epithelial gene program that showed enhanced self‐renewal (i.e., CSC properties) also upregulated genes associated with pluripotency and self‐renewal, including *KLF4*, *MYC*, *SOX2*, *KLF9*, and *LIN28A*, thus linking these abilities to the pluripotency properties observed in iPSCs. Expression of the epithelial determinant E‐cadherin was shown in breast cancer cell lines to be required to uncover stem cell properties such as the formation of mammospheres in nonadherent cell culture 28. This sequential model to explain how tumor cells can migrate and home in distal tissues suggests that cancer cells have inherent cellular plasticity and are able to fluctuate between CSCs and more differentiated states.

## Sox2 Expression Activation as a Tool

We, and others, have demonstrated the role of some of the major pluripotency regulators in the onset of cancer. Sox2 seems to have a prominent role. The *Sox2* gene is amplified and/or its expression is increased in lung and esophageal squamous cell carcinoma (SSC), identifying it as an oncogene for these cancers 29. Also, *Sox2* is expressed in both mouse and human preneoplastic skin lesions and SCC but not in normal epidermis. The deletion of *Sox2* in melanoma or SCC caused regression of tumors, and a number of genes involved in proliferation, stemness, and cell survival are regulated by Sox2 in these tumors, providing further evidence of a role for Sox2 in carcinogenesis 30. Sox2 is also expressed in breast carcinoma mammospheres and is necessary for tumor formation in vivo 31. Using elegant lineage tracing experiments in mouse models of medulloblastoma 32, CSCs were mapped to rare therapy‐resistant quiescent cells that expressed Sox2. Thus, the aberrant activation of Sox2 within a group of transformed cells causes them to shift toward a CSC phenotype, highlighting the plastic nature of neoplastic cells and lining to the mechanisms used to control the pluripotent state. The observation that Sox2 expression is not required for late stages of tumor development suggests that, similar to pluripotency reprogramming, once reprogramming has occurred and the tumor fate has been started (or at least primed), the initiating genetic events might no longer be necessary for the later stages of malignant development 31, 33. Therefore, the initiating lesion could become a passenger mutation.

In addition to increasing our understanding of tumor biology and the roots of cancer, Sox2 activation could be used as a tool to accelerate drug discovery for cancer treatment. After the initial flurry of activity for anticancer stem cell drug development a decade ago, the biology of cancer stem cells has proved complex and difficult to translate into effective therapeutic strategies. Conventional drug screening relies on validated targets for which fast and automated assays are developed. However, to monitor the effect of test compounds on cancer stem cells, targets must be defined. This is, however, cumbersome, because for many tumor indications, cancer stem cell targets have not been clearly defined, with often nonoverlapping combinations of markers defining cell populations with cancer stem cell activities or tumor initiation ability. This most likely reflects the changing nature of the stemness capacity in tumor cells. The evolution of tumor cell populations and the existence of competing programs promoting invasiveness and pluripotency points to a dynamic state in tumor cells characterized by the interconversion of cells with and without stem cell properties, regardless of the actual nature of the driver promoter for plasticity. Aberrant activation of Sox2 in tumor cells might provide this specificity for cancer stem cell‐targeted drug screening. We, and others, have proved that the use of fluorescence protein expression‐based reporters for Sox2 enhancer element activation identifies cells with tumor initiating activity 34, 35, 36, 37. Compounds that are capable of blocking activation of Sox2 in the relevant cell systems might be good candidates for anticancer drug development. These reporter systems could also be used to track CSCs in tumors, study CSC niches, and study the interactions between CSCs and tumor stroma.

## Modeling of Tumor Formation

Few models of human cancer progression are currently available. Primary human cells can undergo cancer progression in mouse xenografts models but require previous transformation with oncogenes different from the endogenous genetic changes found in natural tumors. Patient‐derived human tumor cells are also not a good model, because oncogenic transformation has already occurred, making it difficult to recapitulate the events leading to the generation of CSCs in the first place. Reprogramming primary cancer cells to pluripotent states could be a useful tool to “normalize” tumor cells and then capture the early stages of tumor progression, regardless of the controversy regarding whether the neoplastic state is itself reversible, as only certain cancer genomes or cell types are amenable to this manipulation 38, 39, 40, 41. Small compound screening for drugs that can substitute the expression of pluripotency factors 42 might be used to reveal new drugs able to reprogram cancer cells to a pluripotent state, preventing tumor spread from rapid proliferating nonstem cells. However, methods to control this stem cell population must be set in place in order for this therapy to be successful. Nevertheless, partially reprogrammed tumor cells can be induced to differentiate into disease‐affected lineages, resulting in an unprecedented tool to model tumorigenesis and understand the role of specific oncogenic mutations in that precise tumor type and allowing patient‐specific tumor models. This is very relevant for cancers for which no cellular model is available, such as pancreatic ductal adenocarcinoma. Thus, combining pluripotency reprogramming and animal models demonstrated the continuum from a premalignant phenotype to malignant progression 41, although just one of nine patient samples could be fully reprogrammed, evidencing the intrinsic barriers that transformation imposes on reprogramming. The democratization of genome‐editing technologies within the past few years has made it viable to generate human disease cellular models with greater efficiency. Correction of specific genetic alterations in the genomes of reprogrammed tumor cells would allow one to directly connect genotype and phenotype to establish causality (e.g., for validation of new cancer gene candidates as markers of premalignancy) in an undifferentiated cancer background during its evolution toward more differentiated states. Novel biomarkers for the early detection of cancers might be discovered using this approach, maximizing the possibilities for curative interventions.

The design of new animal models that consider the reprogramming capacities of the oncogenes (e.g., the ones referred to in 43) is necessary to mimic human disease and generate models useful for the development of new therapeutic approaches. Built‐in CSC transcriptional reporters would help to track the evolution of CSC populations during the in vivo evaluation of the next generation of antitumor drugs and contribute to the development of more effective chemotherapy regimens targeting both the bulk of the tumor and the CSC compartment.

## Tools for Ensuring Biosafety of Cell‐Based Therapies

**Figure 1:**
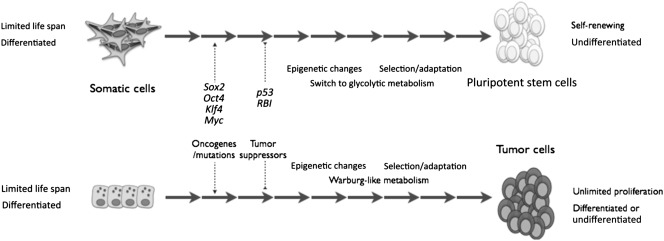
Schematic representation of the parallel processes of pluripotency reprogramming and tumorigenesis from a mechanistic viewpoint. Primary cells of diverse origins are transduced with pluripotency factors or oncogenes that, upon inhibition of tumor suppressors, extensive epigenetic remodeling, and a switch to a glycolytic metabolism, render clones or colonies with embryonic stem cell‐like morphology that are selected manually until fully pluripotent (self‐renewing and undifferentiated) colonies are established. In the case of tumor formation, selection for self‐renewing and undifferentiated (in terms of tumor cell potential) occurs naturally in the organism.

**Figure 2:**
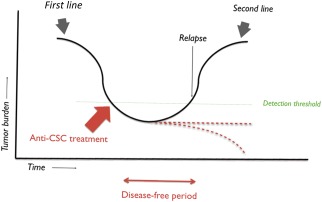
Proposed clinical positioning of anticancer stem cell‐directed treatments. Most oncologic patients will show a response to first‐line treatment; however, if the cancer stem cell compartment is left untouched, resilient cancer stem cells could fuel new tumor growth later on. Therefore, if the cancer stem cell compartment can be controlled after the first line of treatment, relapses and metastases might be prevented. Abbreviation: CSC, cancer stem cell.

As mentioned, some of the multifactorial events involved in the reprogramming and acquisition of stemness properties in pluripotent stem cells can also lead to the generation of malignant tumor cells (Fig. 1). Currently, the major limitation for the use of pluripotent stem cells, and cells derived from pluripotent stem cells, in the clinic is the safety concern, because tumorigenicity remains an unsolved concern for clinical application 44 (Fig. 2). Therefore, a greater understanding of the parallelisms between these two processes might lead to novel approaches to prevent the transformation of cells used as cell therapies, ensuring the biosafety of these treatments. The advances in regenerative medicine and the generation of induced pluripotent stem cells are providing cancer scientists with a new set of tools that will be very useful for the characterization of cancer stem cells.

## Conclusion

The concept of cellular plasticity is challenging how we treat tumors and could explain why tumors relapse. The links found between the acquisition of pluripotency properties and tumor cell plasticity have uncovered novel targets that might be suitable for anticancer therapeutic development, attacking the molecular roots behind the generation of relapses and tumor metastases. More efficient chemotherapy regimens are needed that target the cancer stem cells and the bulk of the tumor composed of more differentiated cancer cells. Conventional cancer therapy has focused on attacking the proliferating cells, with little attention given to tumor cell structure and heterogeneity. Defining the molecular drivers of cancer stem‐like cells will provide avenues for the rational design of cancer treatment regimens aimed at CSCs that, combined with conventional treatments targeting the bulk tumor cells, will provide novel therapies for cancer patients. As complicated as targeted drug design might seem for discovering novel anticancer stem cell‐directed treatments, screening methods based on pluripotency transcriptional reporters could help to identify new drugs targeting cancer stem cells and cellular plasticity. Given the inherent plasticity of cancer cells, it is envisioned that these novel therapeutics would not likely be used as the first line of treatment but would be positioned as maintenance therapy for subsequent relapse prevention after the first line of presumably aggressive therapy has been completed. The arsenal of cytotoxic drugs targeting fast dividing tumor cells is well developed; therefore, combinations with novel drugs that prevent acquisition of stemness properties in cancer cells would ensure a durable anticancer response.

## Author Contributions

J.M.I., J.G., and A.G.M.: manuscript writing, final approval of manuscript.

## Disclosure of Potential Conflicts of Interest

J.M.I. is an employee of Synpromics Ltd. J.G. is an employee of StemTek Therapeutics. A.G.M. is an employee of, and has stock options in, StemTek Therapeutics.
